# Integrating Transcriptome and Genome Re-Sequencing Data to Identify Key Genes and Mutations Affecting Chicken Eggshell Qualities

**DOI:** 10.1371/journal.pone.0125890

**Published:** 2015-05-14

**Authors:** Quan Zhang, Feng Zhu, Long Liu, Chuan Wei Zheng, De He Wang, Zhuo Cheng Hou, Zhong Hua Ning

**Affiliations:** National Engineering Laboratory for Animal Breeding and Ministry of Agriculture Key Laboratory of Animal Genetics and Breeding, Department of Animal Genetics and Breeding, China Agricultural University, Beijing, 100193, China; University of Hong Kong, CHINA

## Abstract

Eggshell damages lead to economic losses in the egg production industry and are a threat to human health. We examined 49-wk-old Rhode Island White hens (*Gallus gallus*) that laid eggs having shells with significantly different strengths and thicknesses. We used HiSeq 2000 (Illumina) sequencing to characterize the chicken transcriptome and whole genome to identify the key genes and genetic mutations associated with eggshell calcification. We identified a total of 14,234 genes expressed in the chicken uterus, representing 89% of all annotated chicken genes. A total of 889 differentially expressed genes were identified by comparing low eggshell strength (LES) and normal eggshell strength (NES) genomes. The DEGs are enriched in calcification-related processes, including calcium ion transport and calcium signaling pathways as reveled by gene ontology (GO) and Kyoto encyclopedia of genes and genomes (KEGG) pathway analysis. Some important matrix proteins, such as *OC-116*, *LTF* and *SPP1*, were also expressed differentially between two groups. A total of 3,671,919 single-nucleotide polymorphisms (SNPs) and 508,035 Indels were detected in protein coding genes by whole-genome re-sequencing, including 1775 non-synonymous variations and 19 frame-shift Indels in DEGs. SNPs and Indels found in this study could be further investigated for eggshell traits. This is the first report to integrate the transcriptome and genome re-sequencing to target the genetic variations which decreased the eggshell qualities. These findings further advance our understanding of eggshell calcification in the chicken uterus.

## Introduction

Calcified eggshells provide protection to egg contents, prevent contamination by microorganisms, and protect the embryo [1]. Eggshells also regulate the exchange of metabolic gases and water, and provide calcium to the embryo. Eggshell damage results in large economic losses each year [2]. Eggshells are composed of two shell membranes, a calcium carbonate layer, and a foamy layer of cuticle [3]. Mineralization of the avian eggshell is a complex process involving the precipitation of calcium carbonate that results from interaction between this mineral and the organic matrix during the nucleation and growth phases [4–7]. Calcification occurs on the shell membrane in the cellular uterine fluid, which contains the inorganic minerals and organic matrix precursors of the eggshell [8]. Calcification is the lengthiest phase of egg formation and occurs in three stages over a 6–22h period following ovulation.

Eggshells contain a proteinaceous matrix of 95% calcium carbonate and 3.5% organic material. Calcium (Ca^2+^) and bicarbonate (HCO_3_^-^) comprise approximately 37.5% and 58% of the eggshell weight, respectively; other minerals are present in trace amounts. Calcium is not stored in the uterus before formation of the shell [5, 9]. Eggshell calcification requires the interaction of numerous processes, including transcellular and/or paracellular transport of Ca^2+^ and secretion of matrix proteins. Many researchers have noted increased uterine Ca^2+^ content during eggshell calcification [10–16]. This supports the hypothesis that a major portion of the Ca^2+^ secreted into the uterus occurs against the electrochemical potential gradient and, therefore, involves active transport. Both Ca^2+^ and HCO_3_^-^ are continuously provided during eggshell formation via blood plasma [17]. Successful interaction of calcium deposition and organic matrix proteins is critically important in the formation of eggshells. Uncovering the key genes that regulate calcium deposition and their mutations would provide an important basis for understanding the eggshell formation process and for altering eggshell qualities.

**Fig 1:**
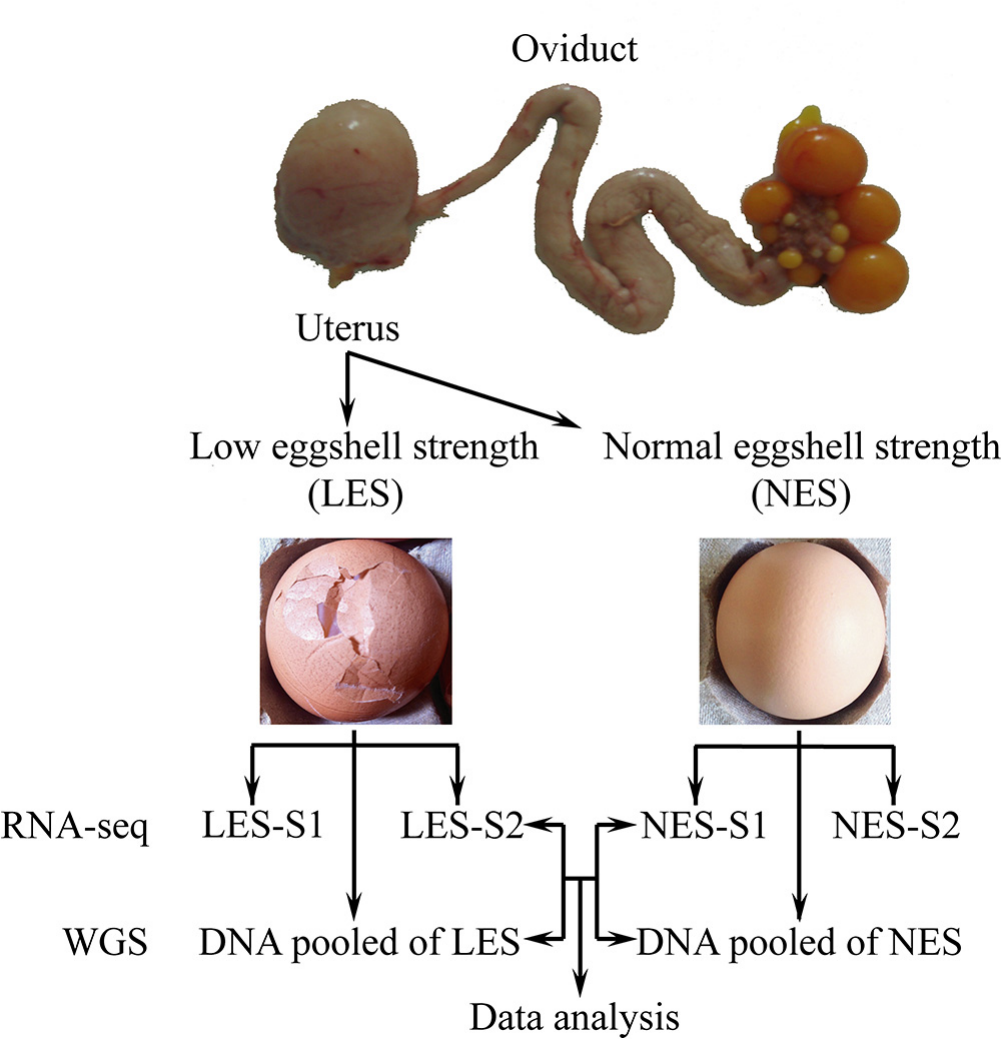
Graphical representation of the experimental strategy. The oviposition cycle of all birds was measured daily, and birds were slaughtered 18 h after ovulation. This stage generally represents the fast growth stage during the eggshell calcification. Individuals from the same sire family that laid eggs with one of two extremely different eggshell qualities were selected. We selected eight birds with normal eggshell strength (NES) and eight birds with very low eggshell strength (LES) for further study. Two biological replicate samples from each group were selected for transcriptome sequencing, and one pooled DNA library for each group was made for whole genome re-sequencing. Finally, we integrated transcriptome and whole genome re-sequencing data to discover the genetic information related to eggshell quality.

A farm-reared Rhode Island White population showed variable eggshell strength. Approximately 15% of individuals laid eggs with very weak eggshells in the same rearing environment as the remainder of the population. This is an excellent model for studying the genetic basis of eggshell formation. In this study, we used systematic whole transcriptome and genome re-sequencing approaches to examine the gene expression and mutations in Rhode Island White hens that lay eggs with extremely different eggshell qualities (Fig 1).

## Materials and Methods

### Ethics statement

Animal experiments were approved by the Animal Care and Use Committee of China Agricultural University (permit number: SYXK 2007–0023). Euthanasia was performed by cervical dislocation in order to quickly obtain the tissue samples to minimize any effect on gene expression changes. All experiments were performed according to regulations and guidelines established by this committee.

### Animals and samples preparation

We observed an exceptionally high proportion of broken eggs in Rhode Island White hens (broken eggshell rate in the laying house >15%). Then, we decided to use this population to study the genetic basis of this phenomenon. All birds were individually reared in cages located in a windowless, air-conditioned poultry house. They were subjected to a cycle of 14 h light: 10 h darkness and were fed ad libitum on a layer diet. All the birds used in this study are healthy. Hens that laid broken eggs were generally evenly distributed across the house.

This population was one of the breeding lines in a breeding farm. The egg production of each bird was recorded daily. The selected birds were representative of the normal production ratio of differing shell quality in this species based on the 25–48 weeks daily records. When the birds were 49 weeks of age, we measured the eggshell qualities of the eggs laid by each bird for 3 days. We measured the eggshell strength and thickness. We carefully checked each hen cage to ensure there was no physical cause for the broken eggs. The experimental design is shown in Fig 1.

### Measurements of eggshell qualities

**Fig 2:**
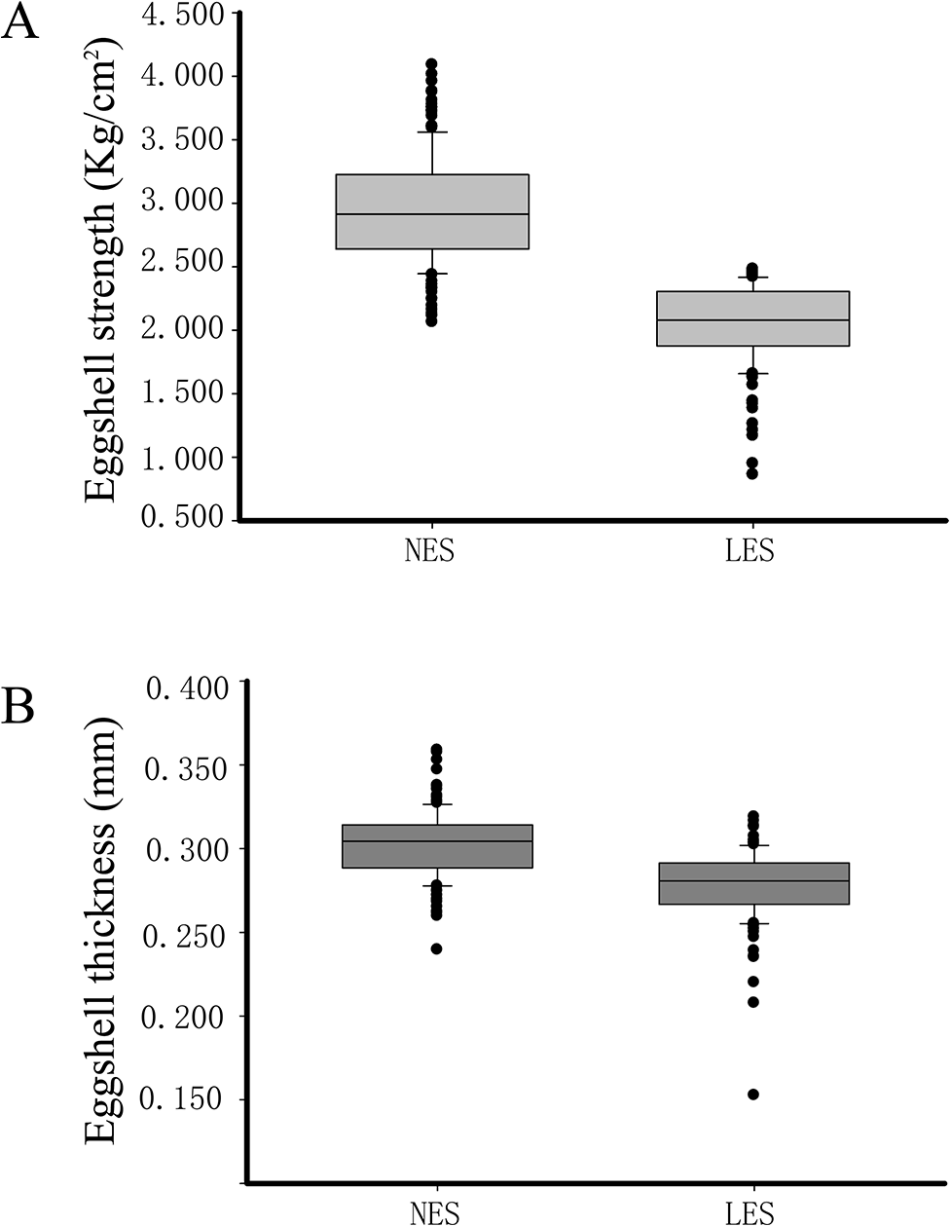
The eggshell strength (A) and eggshell thickness (B) in the normal eggshell strength (NES) group and the low eggshell strength (LES) group. There are 154 and 159 individuals for the NES group and LES group, respectively.

Hens from which 50% or more of the eggs were broken during a 10-day recording period were considered to lay low eggshell strength (LES) eggs. After the first observation stage, the eggshell qualities were measured for each hen in the population. Three eggs were measured for each hen and the average values were considered to represent the eggshell qualities. Eggshell breaking strength and eggshell thickness were measured using the Egg Multi Tester EMT-5200 (Robotmation Co. Ltd). Measurement methods were as previously published [18]. After measurement, descriptive statistics were calculated for all individuals (Fig 2). We chose the individuals that had significantly different eggshell breaking strength and thickness. Finally, we chose eight dam families, each dam family include one normal eggshell strength (NES) bird and one low eggshell strength (LES) bird, for further study (Table 1). The oviposition cycle of all 16 birds was measured daily, and they were slaughtered 18 h after the previous oviposition. This stage generally represents the fast growth stage during eggshell calcification [6].

**Table 1:** Eggshell quality in Rhode Island White hens.

F.	Low eggshell quality			Normal eggshell quality		
	Samples	EST (mm)	ESS (Kg/cm^2^)	Samples	EST (mm)	ESS (Kg/cm^2^)
51	L1	0.263	2.080	N1	0.240	2.722
67^A^	L2	0.247	1.776	N2	0.353	3.776
30	L3	0.282	1.757	N3	0.291	3.072
48	L4	0.250	-	N4	0.283	2.383
43^A^	L5	0.235	-	N5	0.306	3.132
73	L6	0.279	-	N6	0.307	3.188
42	L7	0.255	1.420	N7	0.302	2.890
18	L8	0.275	-	N8	0.313	3.199
	Mean+SD	0.261±0.017**	-	Mean+SD	0.299±0.030**	3.045±0.380

F.: Families. EST: Eggshell thickness. ESS: Eggshell-breaking strength. “-”: Eggshell broken prior to collection.

**: *P*<0.01 (one way ANOVA analysis).

^A^ Four chickens from two dam families were randomly selected for RNA-Seq analysis. Eight equal quantities of DNA samples were mixed to form a pooled DNA for each group.

Uterus tissues were collected from the hens at 18 h after previous oviposition and were snap-frozen in liquid nitrogen and stored at −80°C prior to total RNA extraction. Two individuals were randomly selected from each group for RNA-Seq analysis (Table 1). Blood samples were collected in acid citrate dextrose (ACD) anticoagulant and stored at −20°C prior to DNA extraction. Equal-quantity DNA samples from all eight hens in each group were mixed to form pooled DNA samples.

### RNA-Seq and whole genome re-sequencing

Total mRNA was extracted for sequencing using TRIzol reagent (Invitrogen) following the manufacturer’s instructions. The total mRNA was treated with DNase I and purified with the miRNeasy Mini Kit (Qiagen) according to the manufacturer’s instructions. The quality and concentration of RNA was checked with an Agilent 2100 Bioanalyzer (Agilent) and Nanodrop ND-2000 spectrophotometer (Thermo Scientific). Genomic DNA for whole genome re-sequencing was prepared with a TIANamp Blood DNA Kit (Tiangen) according to the manufacturer’s instructions.

Two biological replicates were generally used in the RNA-Seq related studies [19, 20] as the estimate biological variation highly reliably even when the number of replicates is very small [21]. We used an Illumina HiSeq2000 [22] to perform genome re-sequencing and RNA-Seq. Libraries were constructed following the Illumina Paired-end Sequencing Library Preparation Protocol. A 100-bp paired-end library was constructed for each sample according to the manufacturer’s instructions (Illumina). Library quality and concentration were determined using an Agilent 2100 Bioanalyzer. A standard Illumina base-calling pipeline was used to process the raw fluorescent images and the called sequences. Read qualities were evaluated using the FastQC package (www.bioinformatics.babraham.ac.uk/projects/fastqc/). For genome re-sequencing data, short-reads were trimmed 15-bp from the 3′-end, according the base quality distributions. For RNA-Seq data, short-reads were trimmed 20-bp from the 3′-end according the base quality distributions.

### Reads alignment and mutation analysis for whole genome re-sequencing data

Paired-end short reads were aligned to the *G*. *gallus* reference genome (Ensembl v72, Galgal 4) using the *Burrows-Wheeler Aligner* (*BWA*, *version 0*.*6*.*2*) algorithm with default parameters [23]. SAMTools [24] was used to remove duplicate reads that might have been caused by PCR. To enhance the accuracy of read alignment, aligned reads were realigned at putative SNPs and Indel positions using the Genome Analysis Toolkit (GATK) realigner algorithm (version 2.5.2) [25]. Base quality scores were recalibrated using the GATK recalibration algorithm. The options used for SNP and Indel calling were a minimum 10-read mapping depth, consensus quality of 30, and prior likelihood for heterozygosity value of 0.001.

### Transcriptomic data analysis

Reads were mapped to the *G*. *gallus* genome assembly (Ensembl v72, Galgal 4) using TopHat version 2.0.8 [26]. TopHat RNA-Seq analysis was used with a maximum of 5 mismatches per read. Expression levels of the transcripts were quantified as reads per kilobase per million reads (RPKM) [27]. Genes with RPKM < 0.04 were not included in the analysis. The DESeq [28] algorithm was used to analyze DEGs between LES and NES samples with a *P*-value of 0.05 as a threshold. Ensembl gene IDs from each group were uploaded to the DAVID Functional Annotation Tool and analyzed for gene ontology (GO) and Kyoto Encyclopedia of Genes and Genomes (KEGG) enrichment [29]. The classification stringency was set to the default parameters. Novel isoform detection and differential expression analysis was carried out using the Cufflinks pipeline [30]. Cufflinks transcripts marked as "u" (intergenic transcript), were treated as novel transcripts.

### Genetic mutation annotation

We used SnpEff [31] to annotate mutations and classify mutations into different categories from whole genome re-sequencing and RNA-Seq data. Ensembl (version 72) and dbSNP (ftp://ftp.ncbi.nih.gov/snp/organisms/chicken_9031/VCF/, updated in June 11, 2013) were the source databases used for annotation.

### Quantitative real-time PCR (qPCR) and direct PCR sequencing

Expression of mRNA was verified by qPCR with cDNA from 16 uterine tissue samples. Chicken *β-actin* (GenBank Accession ID: NM_204305) served as a housekeeping gene. Primer 3 Input (v. 0.4.0) was used with default parameters to generate primer pairs for selected genes (Table A in S1 File). Total RNA was extracted from uterine tissue using TRIzol reagent (Invitrogen) following the manufacturer’s instructions. Total RNA (50 ng/μl) was reverse transcribed using M-MLV reverse transcriptase (Promega Corporation) as recommended by the supplier. The qPCR was performed using the SYBR Green Master Mix (Life Technologies) on an ABI 7500 Real Time system (Applied Biosystems, USA). The experiments were carried out in triplicate. The cycling conditions were 95°C for 5 min, followed by 40 cycles at 95°C for 15 s and 60°C for 1 min. A melting curve was obtained at 60 to 95°C for each sample amplified. We used the 2^−ΔΔCT^ method [32] to analyze the relative changes in gene expression from qPCR experiments.

DNA was extracted by TIANamp Blood DNA Kit (Tiangen) according to the manufacturer’s instructions for isolation of genomic DNA from 16 individual blood samples. Direct PCR sequencing is the method of choice for detecting mutations in the target samples. The primers used for PCR amplification are listed in Table B in S1 File. PCR amplifications were performed with 50ng genomic DNA, 10μl PCR Mix (Yeasen, USA), and add to 20 μl with water. Amplification conditions were: 5 min at 95°C, 35 cycles of 30 s at 95°C, 30 s at the *T*_*m*_ of the primers, and 1 min per kb to be amplified at 72°C, followed by 10 min at 72°C. The PCR products were run on a 1% agarose gel (Gene tech, Spain) in 1×TAE buffer. Sequencing was performed with the ABI 3730XL sequencing.

## Results

### Identification of differentially expressed genes and novel isoforms

The total number of reads varied from 37 to 71 million, and 78.09–80.72% of filtered reads were mapped on the reference chicken genome (Table C in S1 File). Among these, 99.4% of the reads mapped to protein-coding genes. Approximately 68% of the reads were mapped to exons (Fig. A in S2 File). Some differences in read counts were detected between biological replicates, especially between NES-S1 and NES-S2. The quantity of total mRNA analyzed was identical in these samples, indicating that the different read counts may have arisen during processing. However, this discrepancy did not significantly affect gene expression analysis because the RPKM values were corrected for the total number of read counts for each sample.

**Fig 3:**
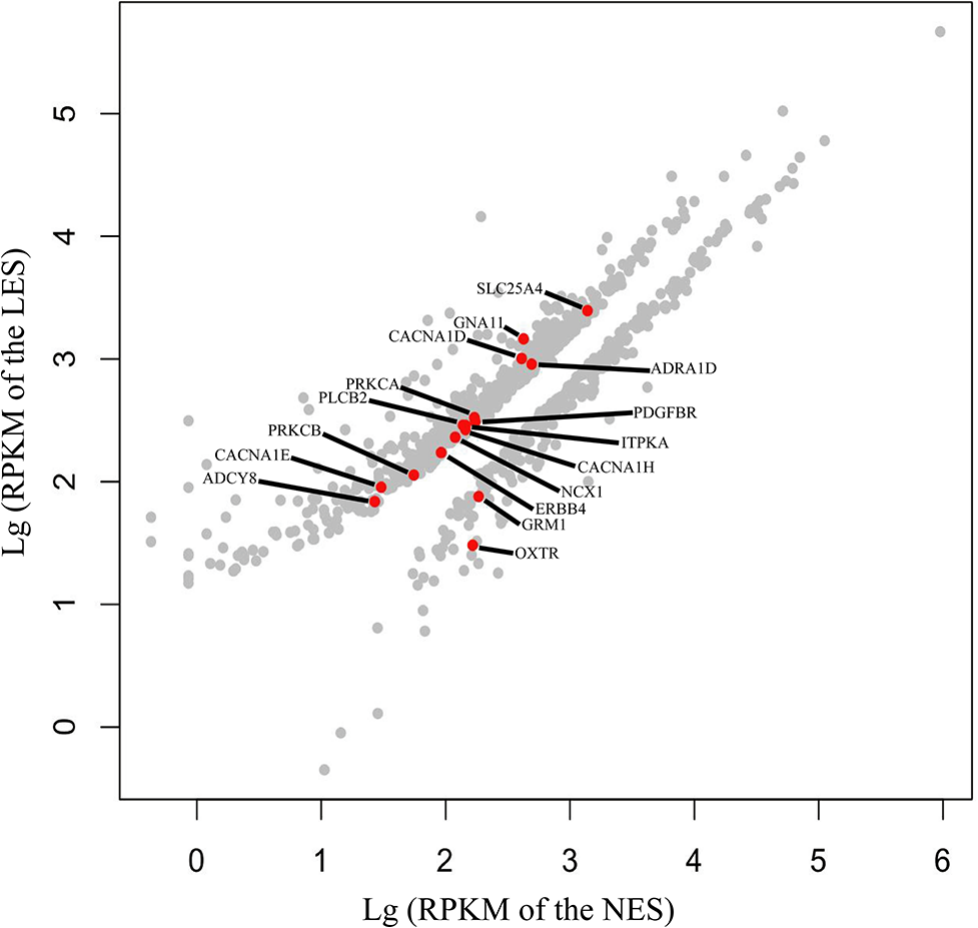
Differentially expressed genes (DEGs) between the low eggshell strength (LES) group and the normal eggshell strength (NES) group. The red spots represent DEGs belonging to a calcium signaling pathway (KO: 04020) (*P* < 0.01).

To identify the DEGs between the two groups, we used DESeq [28] with adjusted *P* < 0.05 for all comparisons. A total of 889 DEGs were detected between the samples (Fig 3, S1 Dataset). Of these, 255 DEGs were significantly up-regulated in NES hens and 634 were significantly up-regulated in LES hens, indicating that uterine genes are differentially expressed in these groups during the calcification of eggshells.

Different isoforms of the gene and novel transcripts were also measured in this study (S2 Dataset). On average 11,092 genes, 21,696 known isoforms and 5,048 novel isoforms were expressed in each sample group (RPKM > 1). There were 118 different promoters and 124 different transcription start sites (*P* < 0.01). However, these promoter and transcription start sites were not significantly different between the two sample groups.

### Functional annotation and pathway enrichment analysis of the DEGs

**Fig 4:**
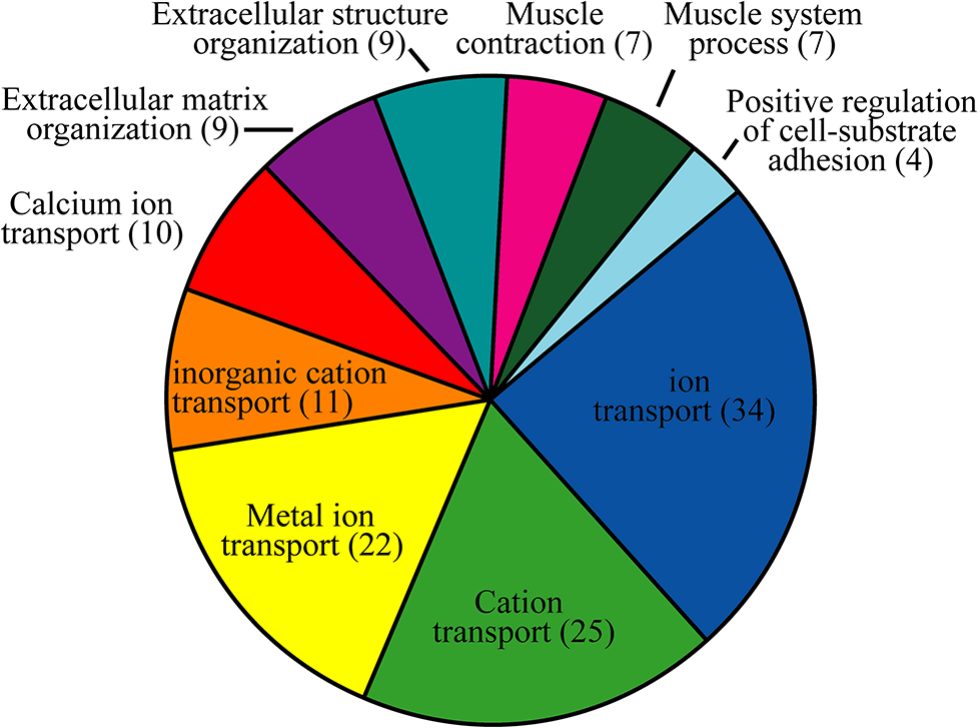
Enriched biological process GO term among differentially expressed genes. Numbers in parentheses indicate the number of differentially expressed genes.

To gain insight into the biological processes regulated during eggshell formation and to determine which processes were coded by DEGs, the DEGs were subjected to GO term enrichment analysis using the DAVID system [29]. We set the threshold P value <0.01 for the GO terms and enrichment score value >1. The GO terms were then classified according to biological process (Fig 4, S3 Dataset). The majority of DEGs were involved in ion transport in the uterus during formation of the eggshell. The GO terms included metal ion transport (GO: 0030001), di- or tri- valent inorganic cation transport (GO: 0015674), ion transport (GO: 0006811), calcium ion transport (GO: 0006816), and cation transport (GO: 0006812). Another important group of DEGs encoded extracellular matrix proteins. Two GO terms were related to the extracellular matrix and were involved in extracellular matrix organization (GO: 0030198) and extracellular structure organization (GO: 0043062). GO terms for muscle contraction (GO: 0006936) and muscle system processes (GO: 0003012) corresponded to seven DEGs for binding actin, ATP, and Ca^2+^. Finally, the GO term for positive regulation of cell-substrate adhesion (GO: 0010811) was related to Ca^2+^ and extracellular matrix binding and cytokine activity.

**Fig 5:**
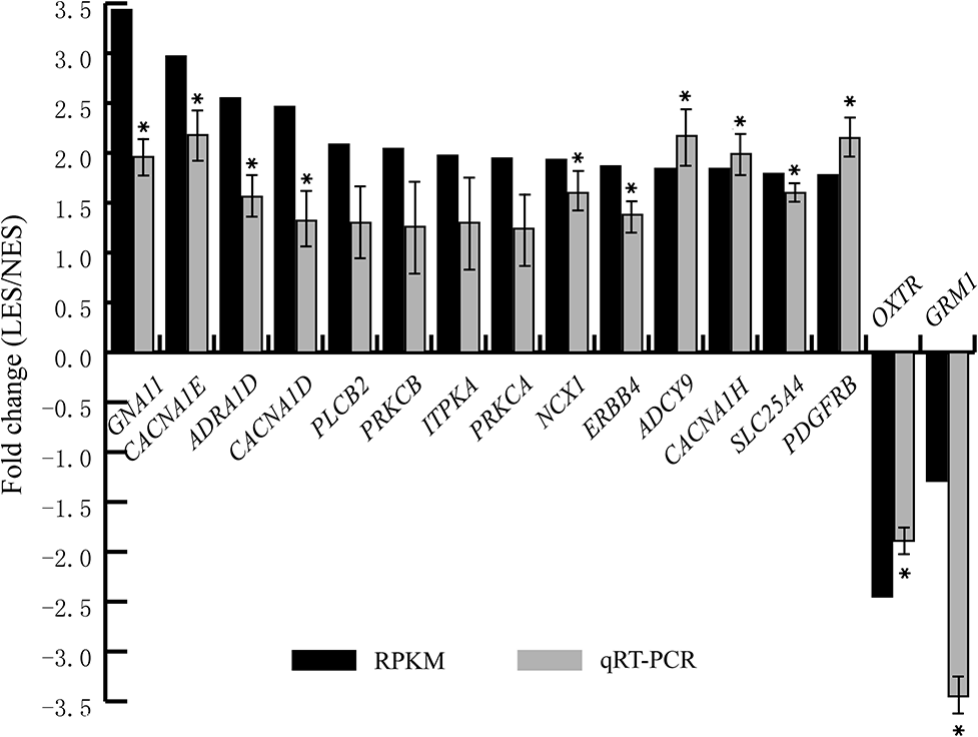
Validation of gene expression in the calcium signaling pathway. The qPCR was performed to quantify gene expression level. The 2^−ΔΔCT^ method was used analyze changes in gene expression relative to chicken *β-actin*. Fold changes between the low eggshell strength (LES) group and the normal eggshell strength (NES) group were calculated for each gene. * represent the significant differentially expressed genes by the qPCR (*P* < 0.05, one way ANOVA analysis). Fold-changes based on RNA-Seq and qPCR were highly correlated (r = 0.71).

KEGG is a knowledge base for systematic analysis of gene functions in terms of the networks of genes and molecules [33]. In this study, KEGG pathway analysis of the DEGs revealed several enriched pathways (*P* < 0.01), including a calcium signaling pathway (KO: 04020), focal adhesion (KO: 04510), extracellular matrix (ECM)-receptor interaction (KO: 04512), and vascular smooth muscle contraction (KO: 04270) (Table 2). The 16 DEGs significantly enriched in Ca^2+^ signaling pathway, and these DEGs belong to G proteins, voltage-dependent Ca^2+^ channels, phosphatidylinositol phospholipase C, Na^+^/Ca^2+^ exchangers, and Na^+^ channels (Fig. B in S2 File). We also confirmed the expression patterns by qPCR which of 16 DEGs in Ca^2+^ signaling pathways (Fig 5), the results of which correlated to the RPKM values estimated by RNA sequencing (r = 0.71).

**Table 2:** KEGG pathway enriched in differentially expressed genes.

Pathway	Pathway definition	P value	Genes
KO: 04510	Focal adhesion	3.88E-05	*TNR*, *MAPK10*, *MYL2*, *TNC*, *ITGA8*, *COL6A3*, *COL3A1*, *PARVA*, *PRKCB*, *PRKCA*, *COL5A1*, *ITGA9*, *THBS2*, *MBSP*, *PDGFRB*, *MAPK9*, *VCL*, *ITGAV*, *ACTN2*, *COL11A1*, *VIN*, *SPP1*, *MET*
KO: 04512	ECM-receptor interaction	2.04E-04	*TNR*, *SV2B*, *TNC*, *ITGA8*, *COL6A3*, *COL3A1*, *COL5A1*, *ITGA9*, *THBS2*, *ITGAV*, *CD36*, *COL11A1*, *VTN*, *SPP1*
KO: 04270	Vascular smooth muscle contraction	1.20E-03	*GNA11*, *ADRA1D*, *CACNA1D*, *CO6*, *PLCB2*, *MRV11*, *PRKCB*, *PRKCA*, *AVTG2*, *ADCY8*, *MBSP*, *KCNMB4*
KO: 04020	Calcium signaling pathway	2.32E-03	*GNA11*, *CACNA1E*, *ADRA1D*, *CACNA1D*, *PLCB2*, *PRKCB*, *ITPKA*, *PRKCA*, *NCX1*, *ERBB4*, *ADCY8*, *CACNA1H*, *SLC25A4*, *PDGFRB*, *GRM1*, *OXTR*

### Whole genome re-sequencing to identify mutations related to eggshell qualities

Whole genome re-sequencing of chickens produced 32.2 and 36.8 Gbp, with coverage depths of 29.82X and 33.77X, for the LES and NES groups, respectively. There were 7,450,661 SNPs in the LES group and 7,588,813 SNPs in the NES group, which corresponded to approximately 8.6 and 7.6 SNP/kb, respectively. Of the SNPs identified in the LES and NES groups, 35.98% and 36.82%, respectively, were novel in comparison to the dbSNP database [34] (Table D in S1 File). SNP density varied and was lowest in sex chromosomes (Z and W chromosomes; avg. 3.78 and 3.90 SNP/kb, respectively) and highest in chromosomes 16 and LEG64 (avg. 12.63 and 13.00 SNP/kb, respectively). SNP density in chromosomes varied from 7.19 to 9.27 SNP/kb (Fig. C in S2 File). We detected 3,671,519 SNPs (484,857 and 563,437 unique SNPs in the LES and NES groups, respectively) for protein-coding genes in the two pooled samples. The number of SNPs and SNP density is similar with previous studies [35–37]. The majority of SNPs were detected in the intronic regions (38.82% for NES group, 38.82% for LES group) and intergenic regions (42.04% for NES group, 42.03% for LES group).

A total of 809,833 and 829,107 Indels were found, corresponding to approximately 0.79 and 0.81 Indels/kb for LES and NES hens, respectively. Of these Indels, 88.83% and 88.90% in the LES and NES groups, respectively, were novel (Table D in S1 File) compared with dbSNP. We detected 508,035 Indels (67,925 and 76,319 unique Indels in the LES and NES groups, respectively) for protein-coding genes in the two pooled samples. Number of detected Indels is also similar with previous report [35, 38]. The majority of Indels were detected in the intronic regions (40.21% for NES group, 40.26% for LES group) and intergenic regions (42.74% for NES group, 42.56% for LES group). Indels found in the exons were deserved in the further analysis.

Non-synonymous or frame-shift mutations may cause an amino acid substitution in the corresponding protein product, thus affecting the phenotype of the host organism. In this study, 1775 instances of non-synonymous and 19 frame-shift mutations were detected in 427 DEGs. The GO terms of the 427 DEGs included di- or tri- valent inorganic cation transport, calcium ion transport, ion transport, metal ion transport, and cation transport. Seven genes were assigned to calcium ion transport biological processing of gene ontology (GO-BP) terms. In the LES group, two common non-synonymous variations were detected in *NCX1*, two frame-shift mutations and one common non-synonymous variation were detected in *PRKCB*, and one non-synonymous variation and one unique non-synonymous variation was detected in *TRPC6*. In the NES group, one non-synonymous variation and one unique non-synonymous variation was detected in *CACNA1H*. *Ovocleidin-116* (*OC-116*) is an important matrix protein that is involved in eggshell calcification. We conducted further validation one non-synonymous mutation of *CACNA1H* gene to test relationship of the SNP and eggshell strength in larger population (Table 3). In our experimental population, we observed that the genotype (AG) has a strong impact on the eggshell strength and thickness (*P* value<0.001, Table 3). According to the RNA-seq and whole genome re-sequencing, 10 non-synonymous variations occurred in *OC-116*, including one and two unique variations in the LES and NES groups, respectively. The SNP mutations in the DEGs of the calcium signaling pathway and matrix proteins are presented in Table 4. The Indel mutations in the DEGs are presented in Table E in S1 File.

**Table 3:** Eggshell qualities of different genotypes for *CACNA1H* gene.

Genotypes	n	Eggshell strength (Kg/cm^2^)	Eggshell thickness (mm)
AG	68	2.572±0.390***	0.300±0.022***
AA	160	2.216±0.513	0.287±0.024
GG	10	2.225±0.591	0.282±0.030

*** represent significant difference in line (*P*<0.001) used one-way analysis of variance.

**Table 4:** Non-synonymous mutations of the differentially expressed genes in calcium signaling pathways and matrix proteins.

Genes	Depth	Position	Ref/new coding	Common	PULES.	PUNES.
*NCX1*	16	3:15742695^R^	Atc/Ttc	*		
*NCX1*	19	3:15742743^R^	Gac/Aac	*		
*CACNA1E*	20	8:5693839^R^	gaT/gaA	*		
*CACNA1E*	16	8:5715790^R^	Acg/Ccg	*		
*CACNA1H*	33	14:5238069 ^R^	cGc/cAc	*		
*CACNA1H*	19	14:5306181 ^R^	tTg/tCg		*	
*OXTR*	14	12:19266364^R^	Gtg/Atg	*		
*PRKCB*	17	14:6644303 ^R^	Tcg/Ccg	*		
*DGKE*	38	18:6239253 ^R^	Aac/Gac	*		
*ITGA8*	33	2:20316822 ^R^	Gca/Aca	*		
*ADCY8*	29	2:140544236^R^	gCg/gTg		*	
*GRM1*	26	3:46087815 ^R^	Gct/Act			*
*GRM1*	27	3:46087816 ^R^	gCt/gAt			*
*ADRA1D*	38	4:87899647 ^R^	aCt/aAt	*		
*PLCB2*	20	5:665344 ^R^	Atc/Gtc	*		
*OC-116*	19	4:45085851 ^R^	cAt/cGt	*		
*OC-116*	19	4:45086211 ^R^	cCc/cTc	*		
*OC-116*	29	4:45086662 ^R^	Gct/Tct		*	
*OC-116*	28	4:45086718 ^R^	aGt/aCt	*		
*OC-116*	26	4:45086821 ^R^	Atg/Gtg			*
*OC-116*	22	4:45087409 ^R^	Cct/Act	*		
*OC-116*	14	4:45087496 ^R^	Ggt/Agt	*		
*OC-116*	34	4:45087542 ^R^	caA/caC			*
*OC-116*	24	4:45087709 ^R^	Acc/Ccc	*		
*OC-116*	21	4:45087750 ^R^	gCt/gTt	*		
*LTF*	27	9:4098717 ^R^	Gtt/Att	*		
*LTF*	19	9:4099069 ^R^	aGg/aAg	*		
*LTF*	23	9:4105617 ^R^	Att/Gtt	*		
*LTF*	26	9:4106072 ^R^	Tca/Gca	*		
*LTF*	22	9:4103017 ^R^	aGc/aCc			*
*SPP1*	20	4:45075410 ^R^	Gtg/Atg	*		

Depth: the number of reads to support the variation. Uppercase letter represent single-nucleotide polymorphism (SNP).

^R^ SNP sites were also detected using RNA-Seq.

PULES: Potential unique low eggshell strength. PUNES: Potential unique normal eggshell strength.

## Discussion

In chickens, eggshell calcification occurs in the hen uterus and is one of the most rapid biological mineralization processes known [5].

We compared gene expression and genetic variation between two different phenotypic groups to facilitate identification of candidate genes associated with eggshell calcification in chickens. Previous global gene expression profiling studies of hen oviducts during sexual maturation and eggshell formation revealed large numbers of DEGs [1, 39]. GO analysis and KEGG prediction showed that these DEGs are involved in several major pathways. The Ca^2+^ signaling system includes a very large set of components, including receptors, channels, and Ca^2+^ pumps and exchangers that can be mixed and matched to create a diverse array of signaling units for delivery of Ca^2+^ signals that have very different spatial and temporal properties [40]. The free intracellular Ca^2+^ concentration is an important link in the excitation-contraction coupling mechanism of vascular smooth muscle [41]. In the present study, 16 DEGs (Table 2, Fig. B in S2 File) were included in predicted Ca^2+^ signaling pathways and 14 DEGs (Table 2) were involved in extracellular matrix (ECM)-receptor interaction, suggesting that these are candidate genes for eggshell calcification in chickens. Previous studies have shown that the most highly represented GO terms are related to genes encoding ion-transport proteins [1, 5, 42]. The chicken uterine model provides a large list of ion-transport proteins that supply Ca^2+^ and HCO_3_^-^ and maintain cellular ionic homeostasis [43].

Five genes (*CACNA1D*, *CACNA1E*, *CACNA1H*, *PRKCB*, and *NCX1*) (Table 2, Fig. B in S2 File, S3 Dataset) are involved in the Ca^2+^ transport and the Ca^2+^ signaling pathway. Expression of the five Ca^2+^ transport genes was up-regulated in the LES group relative to that in the NES group. The *NCX1* gene, encoding a calcium pump and exchanger, is predicted to be present only in the apical membranes of uterine glandular cells [43] and to participate in uterine Ca^2+^ secretion [44]. The role of the transporter is clearly established in mammalian intestines and kidneys [45]. Influx of Ca^2+^ through voltage-dependent Ca^2+^ channels plays a critical role in various biological functions [46]. Seven non-synonymous SNP variations were found in the *CACNA1E*, *CACNA1H*, *PRKCB*, and *NCX1* genes.

Calcium transport in the uterus is associated with the counter-transport of Na^+^ and Cl^-^ [5, 47] and with the secretion of minute masses of shell matrix proteins. In this study, three subunits (*SCNN1A*, *SCNN1B*, and *SCNN1G*) of the Na^+^ channel and the *CLCN2* gene were down-regulated in the LES group as compared to the NES group, and one non-synonymous SNP variation was found in the *SCNN1A* gene. Earlier observations indicate that the *SCNN1* gene family is over-expressed in the uterus; expression of *SCNN1A* is higher in older hens compared to younger hens and *SCNN1B* variations are associated with eggshell strength [48]. The *SCNN1G* gene is over-expressed during shell calcification relative to *SCNN1A* and *SCNN1B*, suggesting its involvement in shell calcification in the uterus [43]. The CLCN2 channel, a member of the CLCN (Cl^-^ channel) family, is thought to participate in various functions such as regulation of cardiac activity [49, 50]. Thus, our study suggests that those ion exchangers and channels may lead to changes in calcium carbonate deposition during eggshell calcification in the uterus.

Lots of QTL regions affecting eggshell thickness have been detected by previous linkage studies and they distribute on GGA1, GGA2, GGA5, and GGA7 [51–54]. Some candidate genes for eggshell thickness were also identified on GGA2, GGA4, GGA8 and GGA9[55, 56]. About 64 DEGs were located in the 15 QTL regions that are related with eggshell strength, and 36 DEGs were located in the 3 QTL regions which are related with eggshell thickness (S4 Dataset). Among those DEGs that had been confirmed in the related QTL regions, *CACNA1D*, *GNA11* and *OXTR* were found in calcium signaling pathway.

Eggshells consist of minerals associated with an organic matrix that is composed of proteins, glycoproteins, and proteoglycans. Proteins specifically produced in the uterus are more likely to play a role in eggshell formation than those produced elsewhere [43]. Different organic matrices participate in the three stages of eggshell calcification [17].

Recently, a high-throughput tandem mass spectrometry approach allowed the identification of >500 eggshell matrix proteins [57]. In our study, expression of *OC-116*, osteopontin (*SPP1*), and ovalbumin was down-regulated in the LES group as compared to the NES group. Ovalbumin presence in uterine fluid is predominant at the initial stage of eggshell formation [17] and ovocleidin-116 is a major component of the chicken eggshell matrix observed throughout the palisade layer and most abundant in uterine fluid during the intense eggshell calcification phase [58, 59]. These proteins are likely to play a fundamental role in eggshell formation since it potentially modifies calcite crystal growth *in vitro* [5, 6, 60]. Our results indicated that *OC-116* and ovalbumin gene expressions positively correlated eggshell calcification during the intense eggshell calcification phase. We also found that expression of ovotransferrin (*LTF*) was up-regulated in the LES group as compared to the NES group. The *LTF* protein is observed in the eggshell [61] and in the uterine fluid, especially at the initial stage of eggshell formation. Calcium carbonate crystals grown *in vitro* in the presence of purified *LTF* showed large modifications of the calcite morphology [62]. The mechanism of the *LTF* on the calcite morphology and final eggshell ultrastructure needs to be further studies. These observations indicated that these differential expression genes might be one of the reasons for the eggshell quality variations. These matrix proteins are, therefore, good choice for the study of any relationship between eggshell matrix proteins and shell quality. Ten, one, and five non-synonymous mutations were detected in *OC-116*, *SPP1*, and *LTF*, respectively. Organic matrix proteins involved in eggshell formation have been identified [6] and polymorphisms in eggshell organic matrix genes have been associated with eggshell quality [55]. The extracellular matrix genes, *ACAD* (carbohydrate binding), *ITGA8* (metal ion binding), *LGALS3* (carbohydrate binding), and *COL11A1* (extracellular matrix binding) were differentially expressed between the two groups of the present study.

We sequenced eight predicted group-specific mutations using Sanger-sequencing for each individual to verify the results of whole genome re-sequencing. However, the results are not fully supported by Sanger sequencing. Inconsistence of the RNA-Seq, whole genome re-sequencing and direct PCR sequencing results were found in this study. All eight mutations for further examined, which discovered by whole-genome resequencing, were also detected by Sanger sequencing. However, examined eight predicted group-specific mutations are not the real group-specific mutations after further validating. Predicted group-specific mutations were found at low frequencies in the control group. We filtered out low frequency mutations when analyzing group-specific mutations, however, this method introduced false-positive mutations which were found in these predicted sites (Fig. D in S2 File). Pooled whole genome resequencing results are good at finding mutations, but not good at discovering the group-specific mutations if resequencing coverage is not high enough, especially in difficulty to locate rare mutations. This needs to be further studied in the whole genome resequencing.

In summary, we analyzed DEGs and mutations from Rhode Island White hens with different eggshell qualities. The identified DEGs are involved in calcium ion transport and calcium signaling pathways. Some important matrix proteins, such as *OC-116*, *LTF* and *SPP1*, were also differentially expressed in the uterus between two groups during eggshell formation stage. Thirty-one non-synonymous mutations (as identified using RNA-Seq and WGS) were found in the DEGs. Genes which involved in ion transport and matrix proteins might be the major genetic factors affecting the eggshell strength in the studied Rhode Island White hen population. The genotype (rs312834462) of *CACNA1H* gene has a strong impact on the eggshell strength and thickness. This is the first report to integrate the transcriptic and genomic re-sequencing to target the genetic variations which decreased the eggshell qualities. Our study enhances our understanding of genetic variation in eggshell quality in relation to eggshell formation.

## Supporting Information

S1 Dataset Total differentially expressed genes (DEGs) between the low eggshell strength (LES) group and normal eggshell strength (NES) group. (XLSX)

S2 Dataset Different isoforms of the gene and novel transcripts. (XLSX)

S3 Dataset Gene ontology (GO) terms according to biological process. (XLSX)

S4 Dataset Differentially expressed genes (DEGs) in the QTL regions which are related with eggshell strength and eggshell thickness. (XLSX)

S1 File Table A. Primers used for qPCR of calcium signaling pathway genes. Table B. Primers used for direct PCR sequencing of the potential group-specific mutations. Table C. RNA sequencing of eggshell quality in Rhode Island White hens. Table D. Whole genome re-sequencing of eggshell quality in Rhode Island White hen. Table E. Frameshift mutations in the differentially expressed genes in KEGG pathway.
(PDF)

S2 File Fig A. **Distribution of mapped reads to different transcript types and gene regions.** Top graph (A and B) indicates the proportion of transcripts belonging to different RNA species. Numbers within pie chart indicate number of reads while numbers in parenthesis indicate number of transcripts. Bottom graph (C) shows the read distribution within protein coding genes. **Fig. B. Differentially expressed genes (DEGs) in calcium signal pathway.** The red markers represent the DEGs in LES compared with NES. NCX was a *NCX1* gene; ADCY was a *ADCY8* gene; CaV1 was a *CACNA1D* gene; CaV2 was a *CACNA1E* gene; CaV3 was a *CACNA1H* gene; GPCR included *ADRA1D*, *GRM1* and *OXTR* genes; PTK included *PDGFRB* and *ERBB4* genes; Gq was a *GNA11* gene; PLC *β* was a *PLCB2* gene; ANT was a *SLC25A4* gene; IP3 was a *ITPKA* gene and PKC included *PRKCA* and *PRKCB* genes. Red marker circle of Genes in KEGG pathway also involved in biological process (BP) term of calcium ion transport. **Fig. C. Graphical representation of two pooled samples genomes in a Circos plots.** Form outside to inside of circles represent chromosome, density of total genes within the chromosome that from red to blue represent for gene density which is from high to low, mapping depth of NES and LES, SNP mutations in the chromosome of NES and LES, Indel mutations in the chromosome of NES and LES. A’s chromosmes are from 1 to 15 based on Mb and B’s chromosomes are from 16 to 32 based on 10 Kb, including sex chromosomes. **Fig. D. Direct PCR sequencing detected mutations of *CACNA1H* and *LTF* gene from the RNA-seq and whole genome re-sequencing.** The results indicated that potential unique SNPs for each group by RNA-seq and genome re-sequencing were not unique SNPs, but the SNP frequencies have difference for each group. The SNP of *CACNA1H* gene is rs312834462, and *LTF* gene’s SNP is rs10724671.
(PDF)
