# Supplementary material for: Integrating Transcriptome and Genome Re-Sequencing Data to Identify Key Genes and Mutations Affecting Chicken Eggshell Qualities
Source: PLoS One. 2015 May 14;10(5):e0125890. doi: 10.1371/journal.pone.0125890 (PMC4431873; doi:10.1371/journal.pone.0125890)
Supplement: S1 File — Primers used for qPCR of calcium signaling pathway genes. Table B. Primers used for direct PCR sequencing of the potential group-specific mutations. Table C. RNA sequencing of eggshell quality in Rhode Island White hens. Table D. Whole genome re-sequencing of eggshell quality in Rhode Island White hen. Table E. Frameshift mutations in the differentially expressed genes in KEGG pathway. (PDF) [file pone.0125890.s005.pdf]

Table A. Primers used for qPCR of calcium signaling pathway genes

Gene symbol	Forward primer	Reverse primer
CACNA1E	AGCAGCGACAACACCTACAA	GATGTCAGGGGACAGCAAGT
ERBB4	TACACGTACGGGGCTTTTTG	GTCAGTGCAGGGCTTACACA
PLCB2	ATGGTGGACCTCACTTTCCA	GGTGTACCGAGAAGCGTTGT
GRM1	GAGATGCCCCAGGAAGGTAT	CGGACCATTCCACTCTTGTT
CACNA1H	CTGGAAGGATGGGTTGACAT	GCTATCACAACCAGGCACAA
ADCY8	TGTCTTGCCACGGTTTGTAG	CGTCTGCAAAAAGAATGCTG
PDGFRB	GGCAATGAGCTGGTCAACTT	GATGAGGATGGAACGGATGT
PRKCB	CTCACTGCTGTATGGCCTCA	TCGGCCCTGATGTATATCCT
OXTR	TCTTCTTCGTGCAGATGTGG	GCATGTAGATCCAGGGGTTG
SLC25A4	CAGATCACAGCGGAGAAACA	CGGATGACGTTGGCTAGATT
NCX1	AGCAAGGAGGAGGAAGAACG	ATGAGCTTGTCCACCGTGTT
PRKCA	CCCAACCTTCTGTGACCACT	GTGTGATCCATGCCACAGAG
ITPKA	TTCAGCTGGATGATTTGCTT	TGGTTTCTCACGGGCTTTAG
CACNA1D	AAAGCCCTAAGAGCCTTTCG	AGGGGAACCATGGCTTTTAT
ADRA1D	GGGGTCTTCTTGGCTGTCTT	AGTTGGTGACTGTCTGCAGGT
GNA11	TCTGCCAACTCAGCAAGATG	TCTGATCGTTGGCCTCCTAC

Table B. Primers used for direct PCR sequencing of the potential group-specific mutations

Gene symbol	position	Forward primer	Reverse primer
CACNA1H	14:5306161	ACAAATCTTGCAGCGAACAGA	GGGTGATAGACTTTGCCTTCC
ADCY8	2:140544236	CTACAAATGAGCCAACAGCC	CTATGCCAACTTTCCTAACC
GRM1	3:46087815	TGTTTTGCAGTGAGCCTGAG	AACATATCAACATCCGTCCA
GRM1	3:46087816	TGTTTTGCAGTGAGCCTGAG	AACATATCAACATCCGTCCA
OC-116	4:45086662	ACCTCTGGCTGGGTCCTGGC	ACAAGAGCTGGAGGCCCCTA
OC-116	4:45086821	ACCTCTGGCTGGGTCCTGGC	ACAAGAGCTGGAGGCCCCTA
OC-116	4:45087521	GGCTACCACCCTCACTCCCA	ATCACTCCAGCAAGCAAAGG
LTF	9:4103017	GCTGGAGGATACCTGTGCTT	TGGGCTCTGTCAGTCTGGAGAA

Table C. RNA sequencing of eggshell quality in Rhode Island White hens

	LES -S1	LES -S2	NES -S1	NES -S2
Read length(bp)	100	100	100	100
Sequencing yield(Gbp)	5.9	6.1	3.8	7.1
Total sequenced reads	59,102,740	61,758,198	37,890,548	71,669,488
Mapped reads	47,708,816	49,410,949	29,590,618	55,989,636
Mapping rate	80.72%	80.00%	78.09%	78.12%

LES: low eggshell strength.
NES: normal eggshell strength.

Table D. Whole genome re-sequencing of eggshell quality in Rhode Island White hens

Statistics	Low eggshell strength	Normal eggshell strength
Read length(bp)	101	101
Sequencing yield(Gbp)	32.2	36.8
Total sequenced reads	312,999,278	355,203,584
Mapped reads	295,310,255	334,432,980
Mapping rate	94.35%	94.15%
Mapping depth	29.82 X	33.77 X
Coverage	94.15%	93.95%
No. of SNP calls	7,450,661	7,588,813
% novel SNP calls	35.98%	36.32%
$\theta_{SNP}(10^{-3})$	8.66	7.63
No. of Indel calls	809,833	829,107
% novel Indel calls	88.83%	88.90%
$\theta_{Indel}(10^{-3})$	0.79	0.81
CNV	2,416	2,944

Table E. Frameshift mutations in the differentially expressed genes in KEGG pathway

Genes	Depth	Position	Old/New	Com.	PULES.	PUNES.
STXBP5L	17	1:79112770	CG/C	+		
TMC3	20	10:11576071 [R]	G/GC	+		
CNGB1	17	11:458088	G/GA			+
ASPN	29	12:3391965 [R]	AC/A	+		
PRKCB	13	14:6644361	C/CG	+		
PRKCB	13	14:6644367	C/CG	+		
SVOP	32	15:6613530	G/GA	+		
CCDC60	36	15:9679408 [R]	T/TC	+		
C4	14	16:53183	T/TG			+
B-G	15	16:346206	CG/C			+
PTGDS	12	17:510123	A/AC	+		
KCNH2	10	2:336742 [R]	CT/C		+	
ENSGALG00000009407	28	2:22762387 [R]	GTGGAAGA/G	+		
JPH2	185	10:5855071	T/TCTGGAG	+		
ENSGALG00000026763	27	22:2458509 [R]	AC/A	+		
SLC20A1	18	22:4025356	G/GA			+
SLC20A1	20	22:4025358	AG/A	+		
WDR27	30	3:40501504	GA/G	+		
HPGDS	32	4:36636020 [R]	A/AT	+		

Superscript letter "R" represent SNP sites were also detected using RNA-seq

Com.: common: PULES: Potential unique LES; PUNES: Potential unique NES
