# Supplementary material for: Integrating Transcriptome and Genome Re-Sequencing Data to Identify Key Genes and Mutations Affecting Chicken Eggshell Qualities
Source: PLoS One. 2015 May 14;10(5):e0125890. doi: 10.1371/journal.pone.0125890 (PMC4431873; doi:10.1371/journal.pone.0125890)
Supplement: S2 File — Distribution of mapped reads to different transcript types and gene regions. Top graph (A and B) indicates the proportion of transcripts belonging to different RNA species. Numbers within pie chart indicate number of reads while numbers in parenthesis indicate number of transcripts. Bottom graph (C) shows the read distribution within protein coding genes. Fig. B. Differentially expressed genes (DEGs) in calcium signal pathway. The red markers represent the DEGs in LES compared with NES. NCX was a NCX1 gene; ADCY was a ADCY8 gene; CaV1 was a CACNA1D gene; CaV2 was a CACNA1E gene; CaV3 was a CACNA1H gene; GPCR included ADRA1D, GRM1 and OXTR genes; PTK included PDGFRB and ERBB4 genes; Gq was a GNA11 gene; PLC β was a PLCB2 gene; ANT was a SLC25A4 gene; IP3 was a ITPKA gene and PKC included PRKCA and PRKCB genes. Red marker circle of Genes in KEGG pathway also involved in biological process (BP) term of calcium ion transport. Fig. C. Graphical representation of two pooled samples genomes in a Circos plots. Form outside to inside of circles represent chromosome, density of total genes within the chromosome that from red to blue represent for gene density which is from high to low, mapping depth of NES and LES, SNP mutations in the chromosome of NES and LES, Indel mutations in the chromosome of NES and LES. A’s chromosmes are from 1 to 15 based on Mb and B’s chromosomes are from 16 to 32 based on 10 Kb, including sex chromosomes. Fig. D. Direct PCR sequencing detected mutations of CACNA1H and LTF gene from the RNA-seq and whole genome re-sequencing. The results indicated that potential unique SNPs for each group by RNA-seq and genome re-sequencing were not unique SNPs, but the SNP frequencies have difference for each group. The SNP of CACNA1H gene is rs312834462, and LTF gene’s SNP is rs10724671. (PDF) [file pone.0125890.s006.pdf]

Supplementary figures

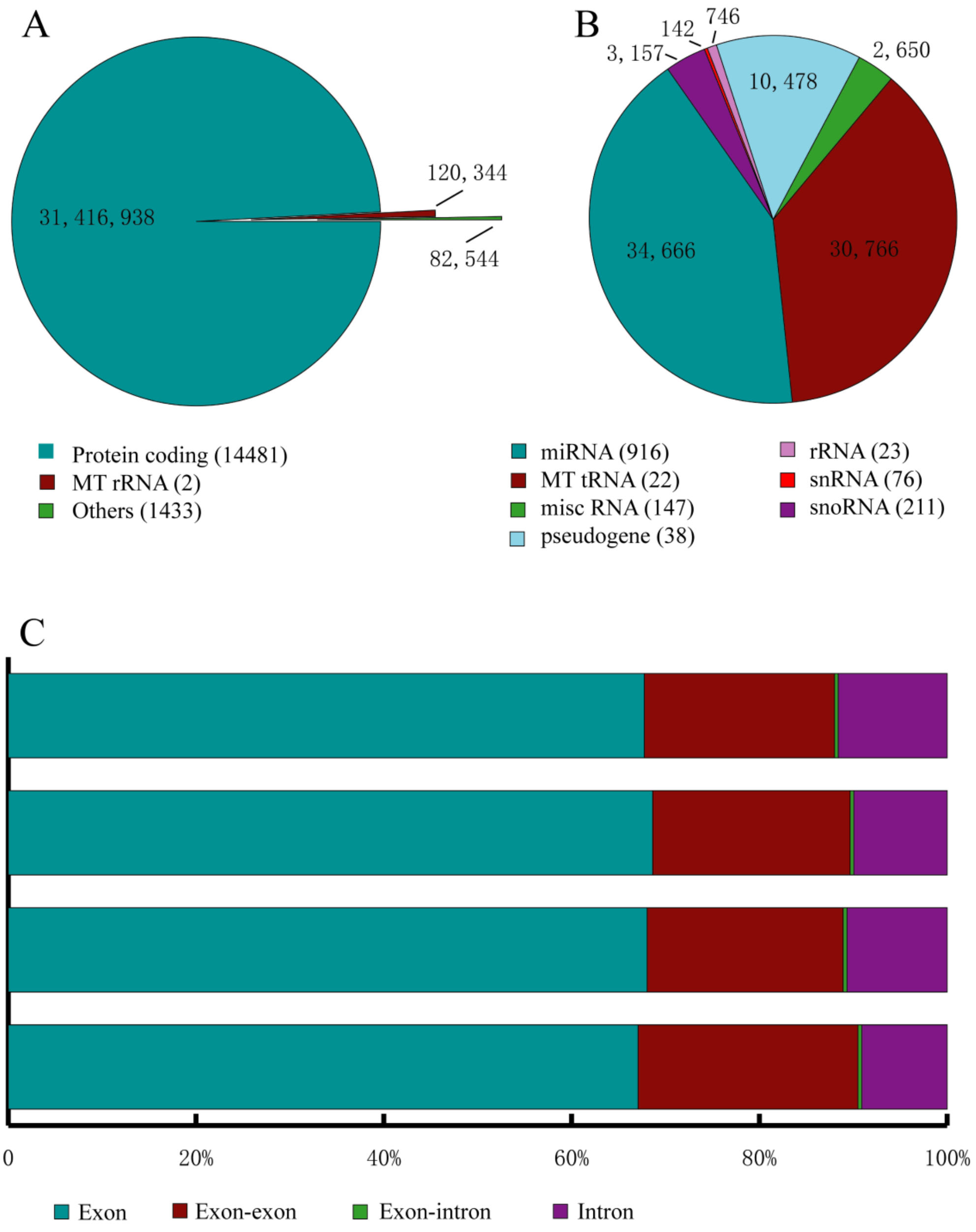

Figure A. Distribution of mapped reads to different transcript types and gene regions. Top graph (A and B) indicates the proportion of transcripts belonging to different RNA species. Numbers within pie chart indicate number of reads while numbers in parenthesis indicate number of transcripts. Bottom graph (C) shows the read distribution within protein coding genes.

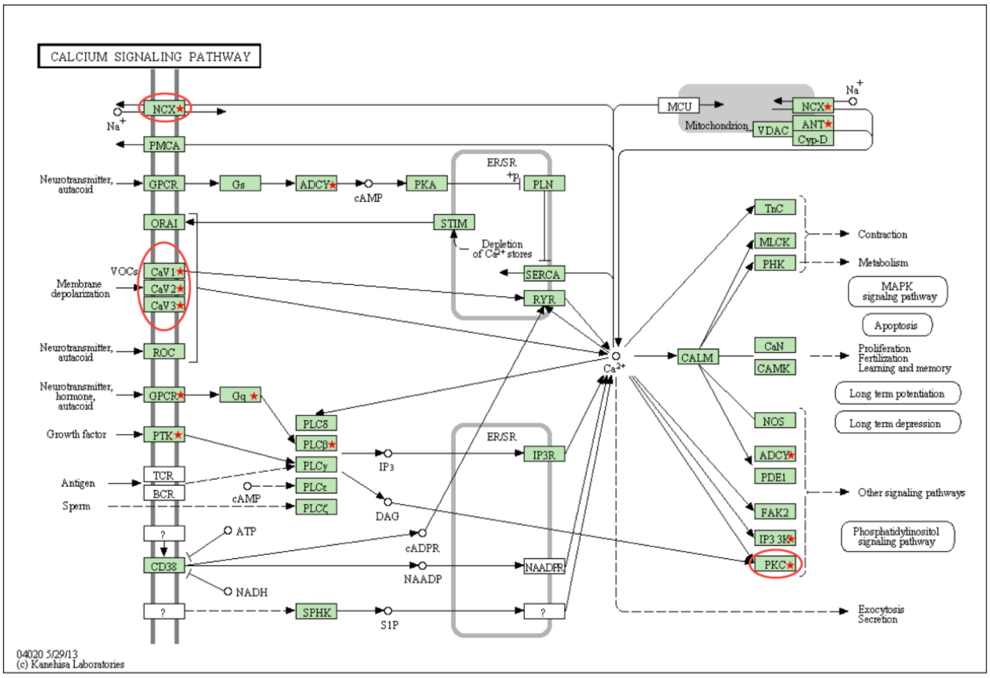

Figure B. Differentially expressed genes (DEGs) in calcium signal pathway. The red markers represent the DEGs in LES compared with NES. NCX was a *NCX1* gene; ADCY was a *ADCY8* gene; CaV1 was a *CACNA1D* gene; CaV2 was a *CACNA1E* gene; CaV3 was a *CACNA1H* gene; GPCR included *ADRA1D*, *GRM1* and *OXTR* genes; PTK included *PDGFRB* and *ERBB4* genes; Gq was a *GNA11* gene; PLCβ was a *PLCB2* gene; ANT was a *SLC25A4* gene; IP3 was a *ITPKA* gene and PKC included *PRKCA* and *PRKCB* genes. Red marker circle of Genes in KEGG pathway also involved in biological process (BP) term of calcium ion transport.

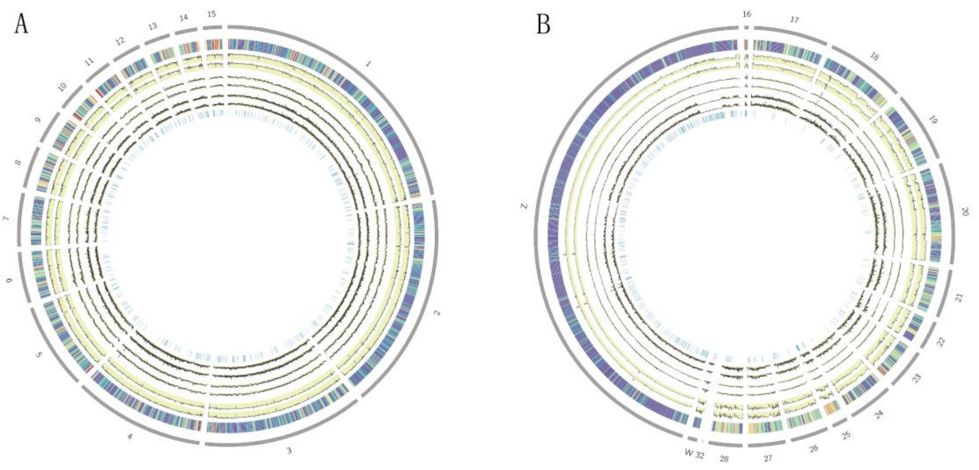

Figure C. Graphical representation of two pooled samples genomes in a Circos plots. Form outside to inside of circles represent chromosome, density of total genes within the chromosome that from red to blue represent for gene density which is from high to low, mapping depth of NES and LES, SNP mutations in the chromosome of NES and LES, Indel mutations in the chromosome of NES and LES. A's chromosmes are from 1 to 15 based on Mb and B's chromosomes are from 16 to 32 based on 10 Kb, including sex chromosomes.

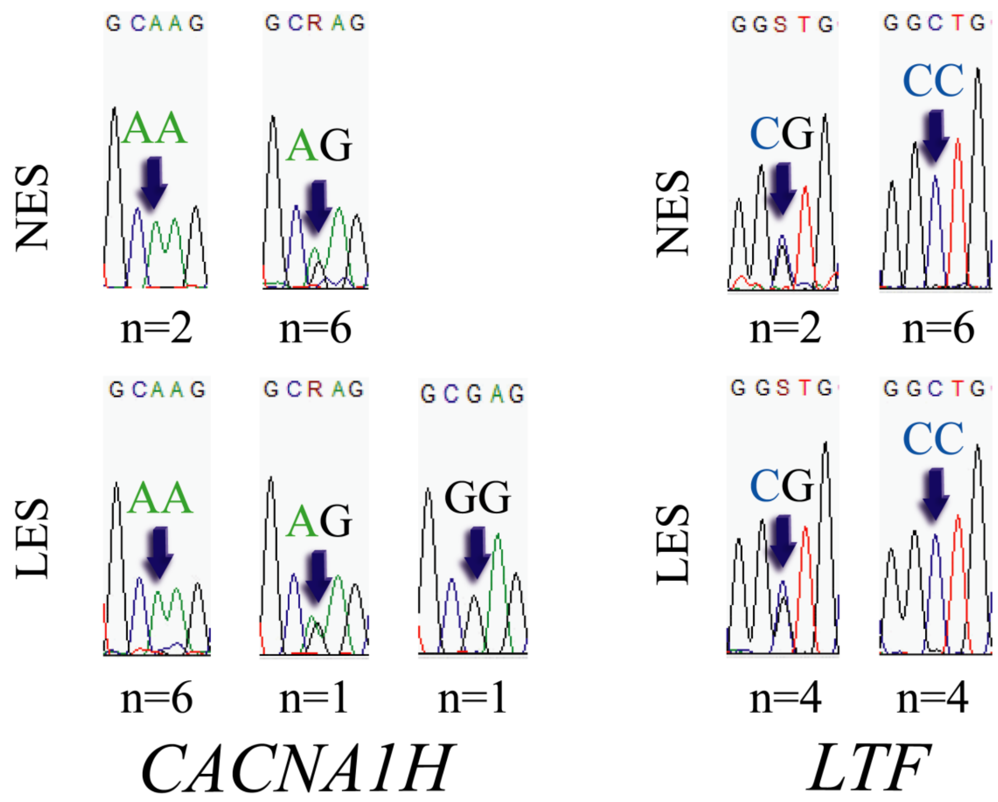

Figure D. Single Nucleotide Polymorphisms (SNPs) of the 16 individuals were detected by direct PCR sequencing. The results indicated that potential unique SNPs for each group by RNA-seq and genome re-sequencing were not unique SNPs, but the SNP frequencies have difference for each group. The SNP of *CACNA1H* gene was rs312834462, and LTF gene's SNP was rs10724671.
